# Supplementary material for: Autophagy of OTUD5 destabilizes GPX4 to confer ferroptosis-dependent kidney injury
Source: Nat Commun. 2023 Dec 18;14:8393. doi: 10.1038/s41467-023-44228-5 (PMC10728081; doi:10.1038/s41467-023-44228-5)

Supplementary Information for

Autophagy of OTUD5 Destabilizes GPX4 to Confer Ferroptosis-Dependent Kidney Injury

Likai Chu, *et al.*

*Correspondance to: junliu@suda.edu.cn

This PDF file includes:

Supplementary Figs. 1 to 7,
Supplementary Tables 1 and 2
Uncropped immunoblots gels for Supplementary Figures 2, 3, 5, 6, and 7

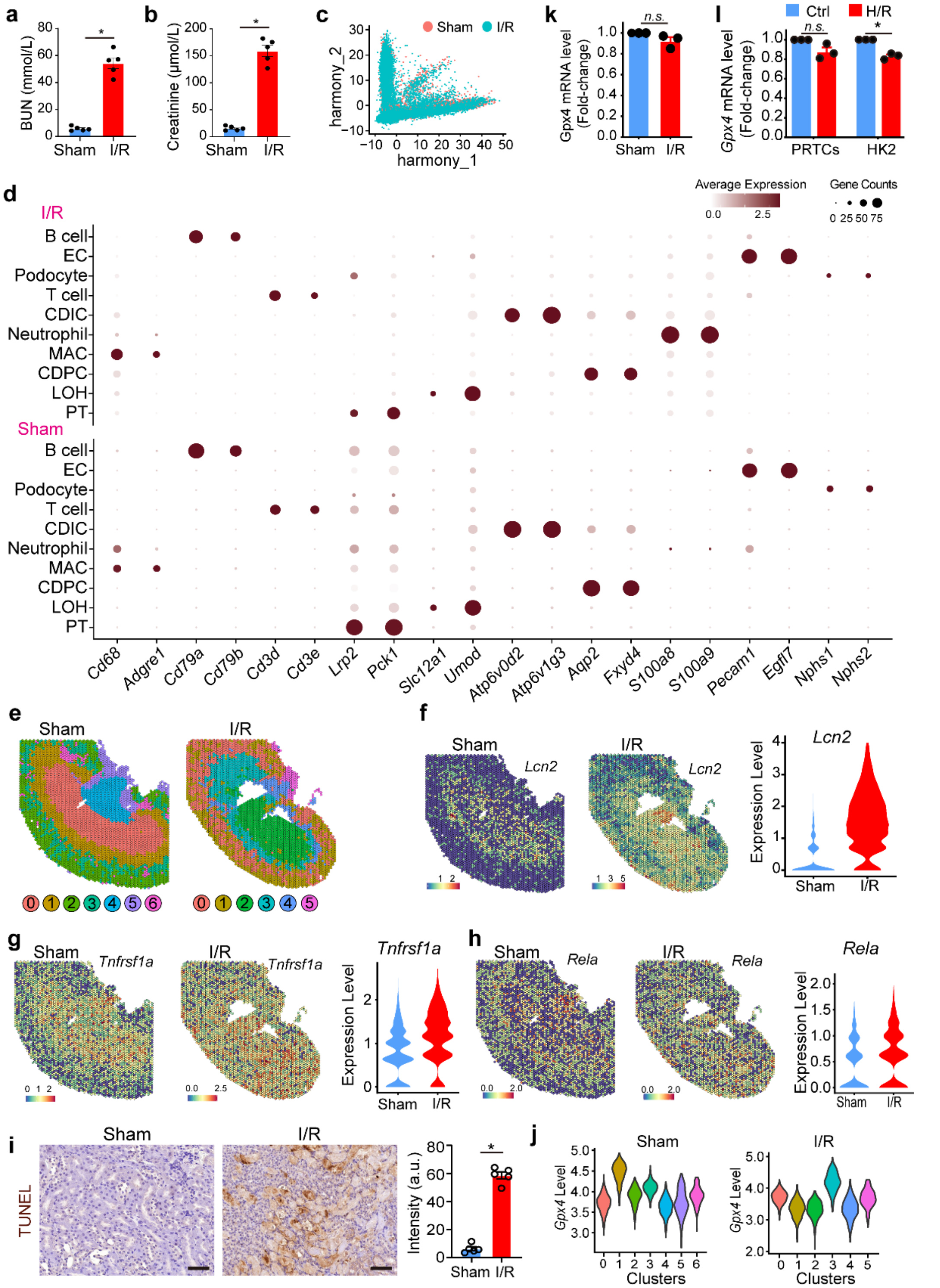

Supplementary Fig. 1. scRNA sequencing and spatial transcriptomics reveal GPX4-mediated tubular cell ferroptosis in I/R-induced AKI. a, b Serum BUN (**a**) and (**b**) creatinine levels of sham or I/R-treated mice. Data are presented as mean ± s.e.m.; statistical significance was determined using an unpaired two-tailed Student's *t*-test, $^{*}p < 0.05$. **c** The batch effect correction of the two samples. **d** The dot plot shows expressions of two representative marker genes of each cell type from scRNA data of Figure 1a. **e** Hematoxylin and eosin (H&E) staining of kidney sections and unbiased clustering of the spatial transcriptome (ST) spots of sham and I/R-treated kidneys. (**f-h**) Spatial feature plots and Violin plots of kidney injury marker gene *Lcn2* (**f**), *Tnfrsf1a* (**g**),

Rela (**h**) in ST spots of sham or I/R-treated kidneys. **i** IHC staining and quantification of TUNEL expression on kidney sections from sham or I/R-treated mice (n =5); scale bars, 50 μm. **j** Violin plots of *Gpx4* expressions in clusters from sham or I/R-treated mouse kidneys. **k, l** mRNA level of GPX4 in mouse kidneys (n = 5 per group), PRTCs, and HK2 cells. Data are presented as mean ± s.e.m.; statistical significance was determined using an unpaired two-tailed Student's t-test, $^{*}p$ < 0.05, *n.s*: no significance.

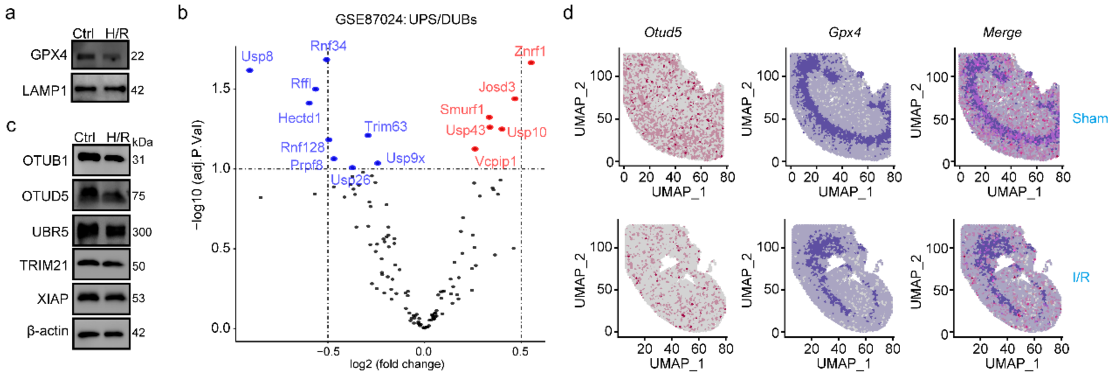

Supplementary Fig. 2. OTUD5 is a GPX4 interacting protein for stabilization in response to I/R. a Immunoblot analysis of GPX4 in the lysosome of cells treated with or without H/R. **b** The volcano plot shows the expression profile of UPS/DUBs-associated genes in the kidney before and after I/R induction (GSE87024), the most dysregulated UPS/DUBs genes were labeled in blue (downregulation) or red font (upregulation). **c** Immunoblot analysis of the indicated proteins in cells treated with or without H/R. **d** Feature plot of *Otud5* and *Gpx4* expression, and their colocalization in ST spots of the two groups.

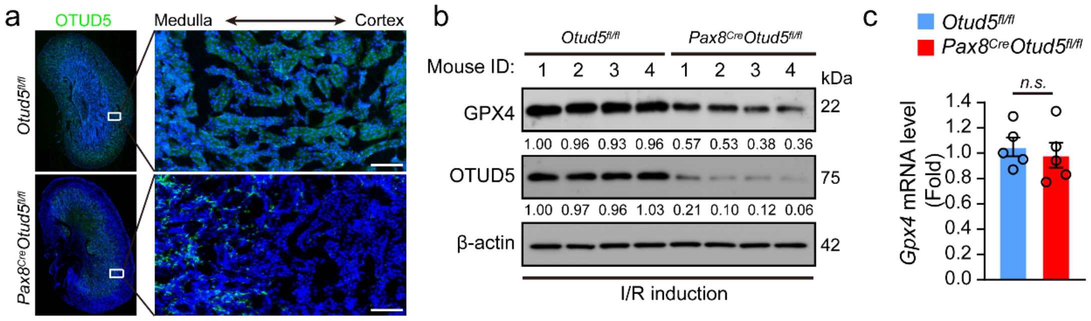

Supplementary Fig. 3. *Otud5* deletion confers kidneys vulnerable to I/R.

a Immunofluorescence analysis of OTUD5 expression in kidney samples from *Pax8*^{Cre}*Otud5*$^{fl/fl}$ mice and their WT littermates (*Otud5*$^{fl/fl}$). **b** 4 to 6-week-old *Pax8*^{Cre}*Otud5*$^{fl/fl}$ (n =4) and their WT littermates (n =4) were subjected to kidney I/R surgery. After 48 hours, kidneys were collected and subjected to immunoblot analysis for OTUD5 and GPX4. **c** The mRNA level of *Gpx4* in *Pax8*^{Cre}*Otud5*$^{fl/fl}$ mice and their WT littermates. All values are presented as mean±s.e.m.; p values were calculated by unpaired two-tailed student's t-test, $^{*}p < 0.05$.

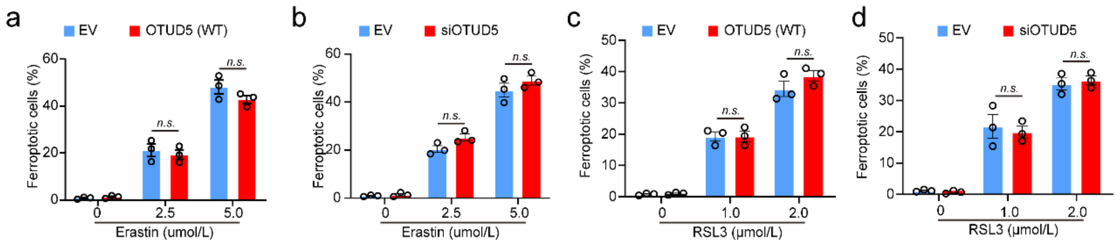

Supplementary Fig. 4. OTUD5's minimal effect on erastin and RSL3-induced ferroptosis in renal tubular cells. a Cell ferroptosis was measured in EV or WT OTUD5-transfected cells in the presence or absence of Erastin with the indicated doses for 24 hours. **b** Cell ferroptosis was measured in siCtrl or siOTUD5-transfected cells in the presence or absence of Erastin with the indicated doses for 24 hours. **c** Cell ferroptosis was measured in EV or WT OTUD5-transfected cells in the presence or absence of RSL3 with the indicated doses for 24 hours. **d** Cell ferroptosis was measured in siCtrl or siOTUD5-transfected cells in the presence or absence of RSL3 with the indicated doses for 24 hours. Data are from three repeated experiments, presented as mean ± s.e.m.; statistical significance between groups as indicated was determined using an unpaired two-tailed Student's *t*-test, *n.s.*: no significance, $*p < 0.05$.

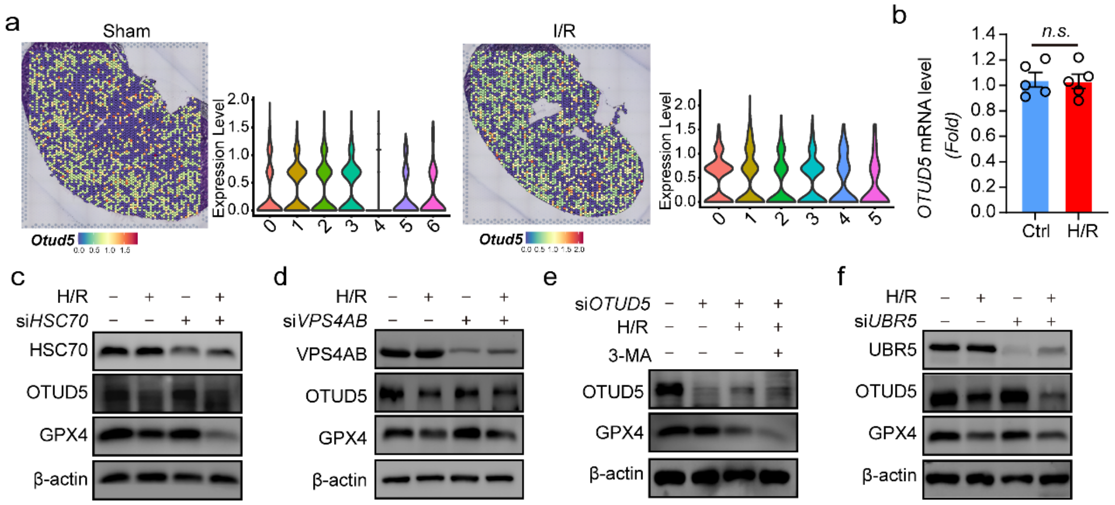

Supplementary Fig. 5. Hypoxia activates autophagy degradation on OTUD5. a Spatial expression pattern of *Otud5* in ST spots and clusters of sham or I/R-treated mice. **b** The expression level of *Otud5* was analyzed in PRTCs treated with or without H/R. **c-f** HK2 cells were transfected with siControl or si*HSC70* (**c**), si*VPS4AB* (**d**) for 48 hours and treated with H/R condition. (**e**) OTUD5-depleted HK2 cells were treated with H/R conditions in the presence or absence of 3-MA. (**f**) HK2 cells were transfected with siControl or si*UBR5* for 48 hours and treated with H/R condition.

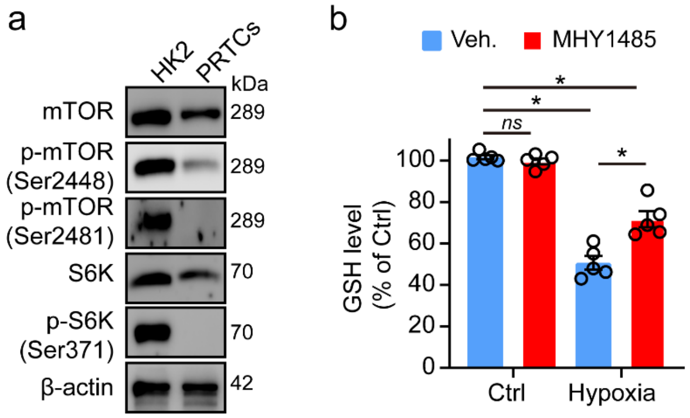

Supplementary Fig. 6. H/R reduces OTUD5 through repressing mTORC1 signaling.

a The protein lysate of HK2 and PRTCs were subjected to immunoblot and the levels of proteins associated with mTOR signaling were analyzed. **b, c** The GSH level (**b**) and GSH/GSSH ratios (**c**) in HK2 cells were determined before and after hypoxia induction. Data are presented as mean ± s.e.m.; Statistical significance was determined using an unpaired two-tailed Student's *t*-test, $^{*}p < 0.05$, *n.s:* no significance.

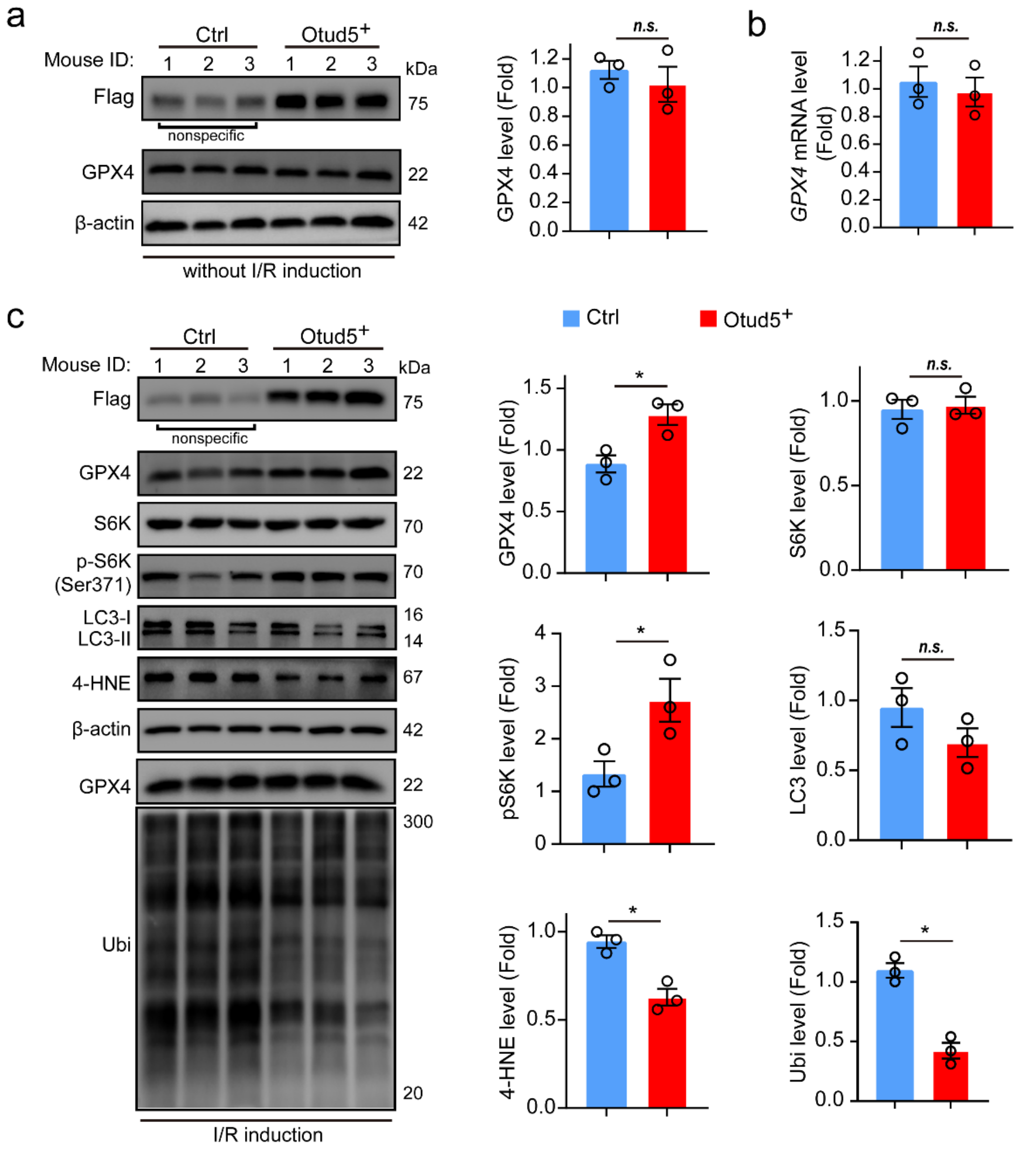

Supplementary Fig. 7. AAV-mediated OTUD5 therapy protects renal function against I/R injury. a 4 to 6-week-old C57BL/6J mice were intravenously injected with a single dose of 3×10^{11} copies of *Otud5*-contained virus. 48 hours after injection, mice were sacrificed and the kidneys were collected for immunoblot. The levels of GPX4 and Flag (OTUD5) were analyzed. **b** The mRNA level of *Gpx4* in the *Otud5*-expressed mice and the control mice (*n*= 3). **c** 4 to 6-week-old C57BL/6J mice were intravenously injected with a single dose of 3×10^{11} copies of *Otud5*-contained virus. 48 hours after injection, mice were subjected to I/R surgery and lived for another 48 hours. After that, mice were sacrificed and the kidneys were collected for immunoblot (*n*= 3). Data are presented as mean ± s.e.m.; Statistical significance was determined using an unpaired two-tailed Student's *t*-test, *n.s:* no significance.

Supplementary Table 1. Reagents and antibodies were used in this study.

**Antibody**	**Supplier**	**Catalog number**	**Clone number**
Anti-Glutathione Peroxidase 4	Abcam	ab125066	EPNCIR144
Anti-Glutathione Peroxidase 4	Abcam	ab41787	
Anti-4 Hydroxynonenal	Abcam	ab48506	HNEJ-2
Anti-F4/80	Abcam	ab300421	EPR26545-166
Anti-OTUD5 Polyclonal Antibody	Invitrogen	PA5-20611	
Anti-OTUD5 Polyclonal Antibody	Proteintech	21002-1-AP	
Anti-OTUD5 Rabbit mAb	Cell Signaling	20087S	D8Y2U
Anti-GPX4 Antibody	Cell Signaling	52455S	
Anti-p70 S6 Kinase Antibody	Cell Signaling	9202S	
Anti-Phospho-p70 S6 Kinase (Ser371)	Cell Signaling	9208S	
Anti-mTOR Rabbit mAb	Cell Signaling	2983S	7C10
Anti-Phospho-mTOR (Ser2481)	Cell Signaling	2974S	
Anti-Phospho-mTOR (Ser2448) Rabbit mAb	Cell Signaling	5536S	D9C2
Anti-DYKDDDDK Tag Rabbit mAb	Cell Signaling	14793S	D6W5B
Anti-Ubiquitin Mouse mAb	Cell Signaling	3936S	P4D1
Anti-Ubiquitin Rabbit mAb	Cell Signaling	20326S	E6K4Y
Anti-LC3A/B Antibody	Cell Signaling	4108S	
Anti-Beclin-1 Antibody	Cell Signaling	3738S	
Anti-Atg5 Rabbit mAb	Cell Signaling	9980S	D5G3
Anti-Hamartin/TSC1	Cell Signaling	6935S	D43E2
Anti-LAMP1 Rabbit mAb	Cell Signaling	9091S	D2D11
Anti-OTUB1 Rabbit mAb	Cell Signaling	3783S	D8F7
Anti-UBR5 Rabbit mAb	Cell Signaling	65344S	D6O8Z
Anti-XIAP Antibody	Cell Signaling	2042S	
Anti-TRIM21 Rabbit mAb	Cell Signaling	92043S	D1O1D
Anti-mouse IgG HRP-linked Antibody	Cell Signaling	7076S	
Anti-rabbit IgG HRP-linked Antibody	Cell Signaling	7074S	
Anti-β-actin Rabbit mAb	Cell Signaling	4970S	13E5
Anti-HSPA8/HSC70	Santa Cruz Biotechnology	sc-7298	sc-7298
Anti-VSP4	Santa Cruz Biotechnology	sc-133122	E-8
Anti-Glutathione Peroxidase 4	Santa Cruz Biotechnology	sc-166437	D-3
Anti-KIM-1 (HAVCR1)	Boster Biological Technology	BA3536	
Normal rabbit IgG	Santa Cruz Biotechnology	sc-2027	
Normal mouse IgG	Santa Cruz Biotechnology	sc-2025	
**Reagent**			

MG132	Selleck	S2619
Bafilomycin A1	Selleck	S1413
RSL3	Selleck	S8155
Cycloheximide	Selleck	S7418
Protein G Plus Agarosse	Santa Cruz Biotechnology	sc-2002
Lipofectamine 3000	Invitrogen	L3000015
MHY1485	Selleck	S7811
7-AAD	Invitrogen	00-6993-50
BODIPY™ 581/591 C11	Thermo Fisher Scientific	D3861
CQ	Selleck	S6999
**Plasmid**		
Mouse_OTUD5	Generalbiol	N/A
Human_His-OTUD5	Generalbiol	N/A
Human_OTUD5 (C224S)	Generalbiol	N/A

Supplementary Table 2. Information of primers used for quantitative real-time PCR analyses in this study.

Gene	Forward (5' to 3')	Reverse (5' to 3')
Mouse_*Gpx4*	CCTCTGCTGCAAGAGCCTCCC	CTTATCCAGGCAGACCATGTGC
Human_*GPX4*	ACAAGAACGGCTGCGTGGTGAA	GCCACACACTTGTGGAGCTAGA
Mouse_*Otud5*	GTTCATGACATTCGGCTT	CACAAGCAAAAGTACCCTA
Mouse_*Lcn2*	TCAAGGACGACAACATCATCTTCT	CTCCAGATGCTCCTTGGTATGG
Mouse_*Havcr1*	TACCTCCACTCCTCCAACATCTA	TGCCAACATAGAAGCCCTTAGTA
Mouse_*Il6*	CCAGAAACCGCTATGAAGTTCCT	TGTGTAATTAGCCTCCGACTTGT
Mouse_*Tnf*	GACCCTCACACTCAGATCATCTT	CCTTGAAGAGAACCTGGGAGTAG
Mouse_*GAPDH*	CATCACTGCCACCCAGAAGACTG	ATGCCAGTGAGCTTCCCGTTCAG
Human_*GAPDH*	ACCCACTCCTCCACCTTTGA	CTGTTGCTGAGCCAAATTCGT

Uncropped immunoblot gels for Supplementary Figure 2

Figure S2A-GPX4

Antibody information: Abcam, ab41787, 1:1000

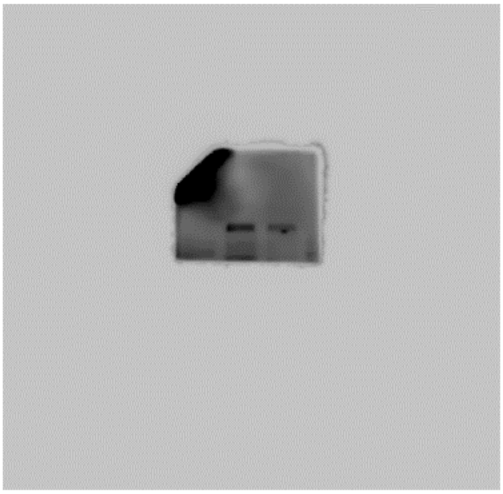

Figure S2C-β-actin

Antibody information: Cell Signaling, 4970S, 1:1000

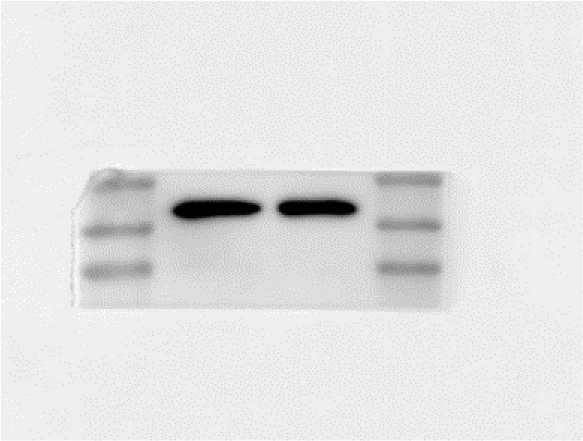

Figure S2A-LAMP1

Antibody information: Cell signaling, 9091S, 1:1000

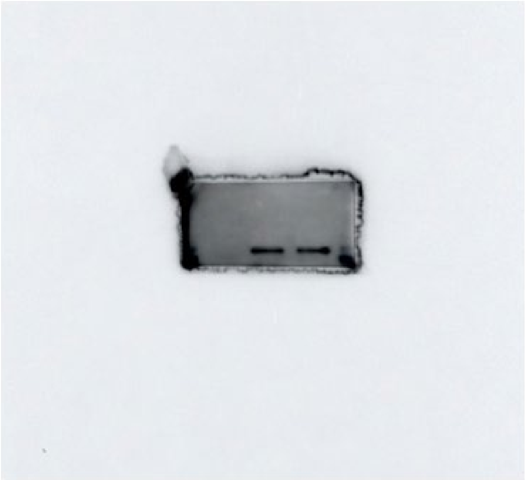

Figure S2C-OTUB1

Antibody information: Cell signaling, 3783S, 1:1000

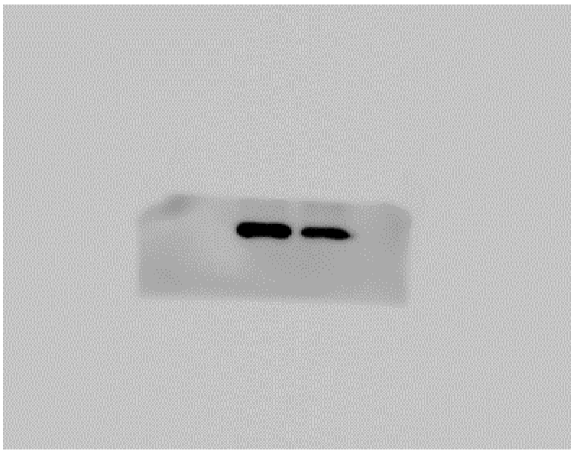

Figure S2C-UBR5

Antibody information: Cell signaling, 65344S, 1:500

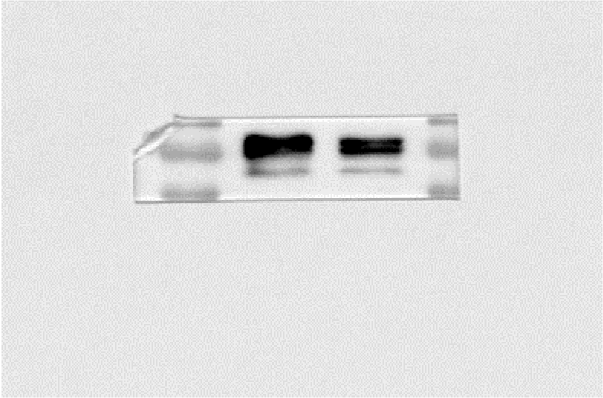

Figure S2C-XIAP

Antibody information: Cell signaling, 2042S, 1:1000

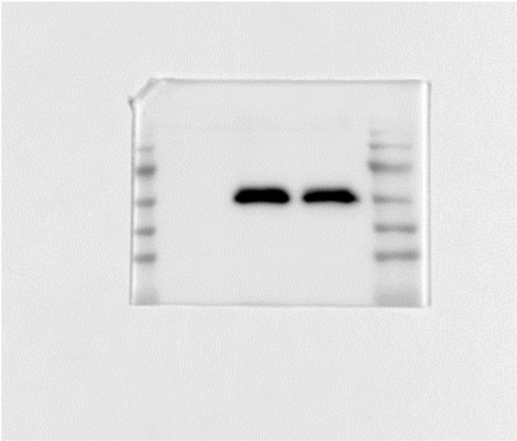

Uncropped immunoblot gels for Supplementary Figure 3

Figure S3B-Actin

Antibody information: Cell Signaling, 4970S, 1:1000

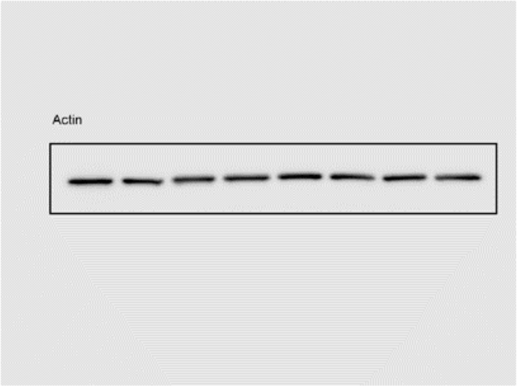

Figure S3B-GPX4

Antibody information: Abcam, ab41787, 1:1000

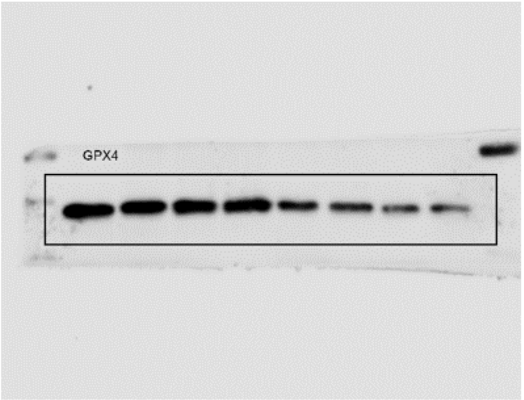

Figure S3B-OTUD5

Antibody information: Cell signaling, 20087S, 1:1000

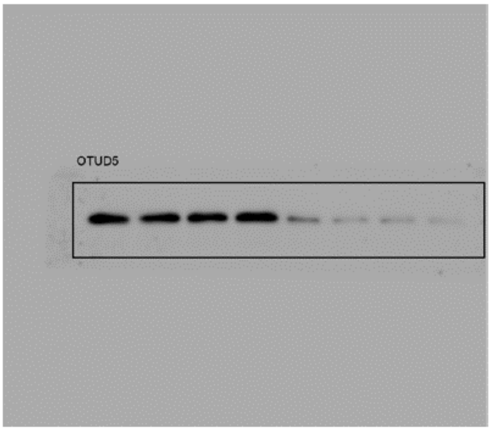

Uncropped immunoblot gels for Supplementary Figure 5

Figure S5E-Actin

Antibody information: Cell Signaling, 4970S, 1:1000

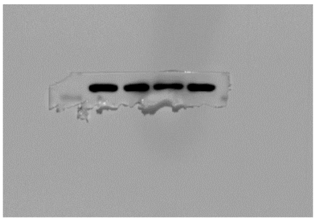

Figure S5C-GPX4

Antibody information: Abcam, ab41787, 1:1000

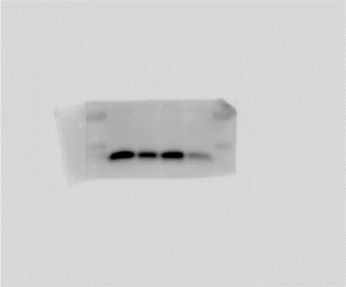

Figure S5C-OTUD5

Antibody information: Cell signaling, 20087S, 1:1000

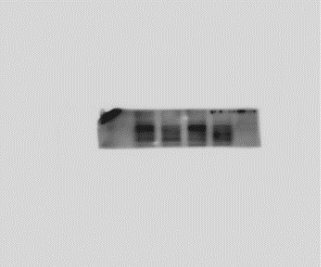

Figure S5C- Actin

Antibody information: Cell Signaling, 4970S, 1:1000

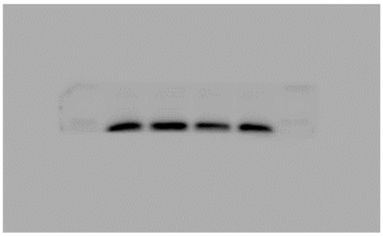

Figure S5D-VPS4AB

Antibody information: Santa Cruz Biotechnology, sc-133122, 1:500

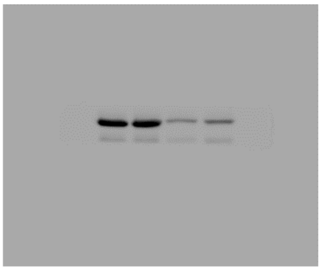

Figure S5D-GPX4

Antibody information: Abcam, ab41787, 1:1000

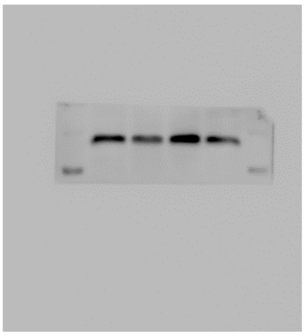

Figure S5D-OTUD5

Antibody information: Cell signaling, 20087S, 1:1000

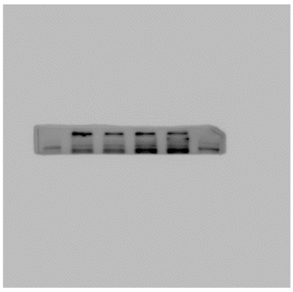

Figure S5D-Actin

Antibody information: Cell Signaling, 4970S, 1:1000

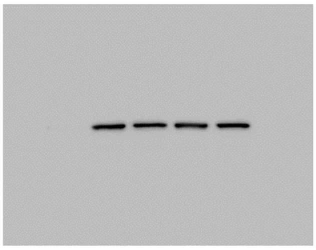

Figure S5E-GPX4

Antibody information: Abcam, ab41787, 1:1000

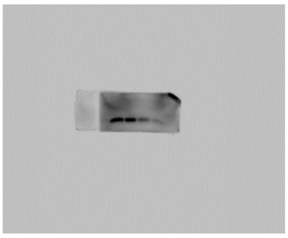

Figure S5E-OTUD5

Antibody information: Cell signaling, 20087S, 1:1000

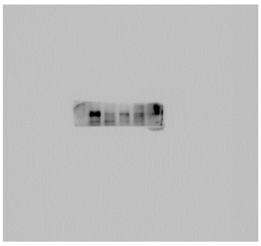

Figure S5C-HSC70

Antibody information: Santa Cruz Biotechnology, Sc-7298, 1:500

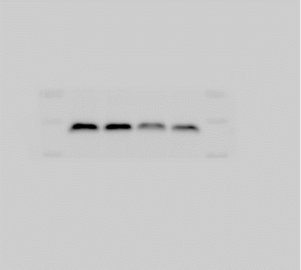

Figure S5F-UBR5

Antibody information: Cell signaling, 65344S, 1:500

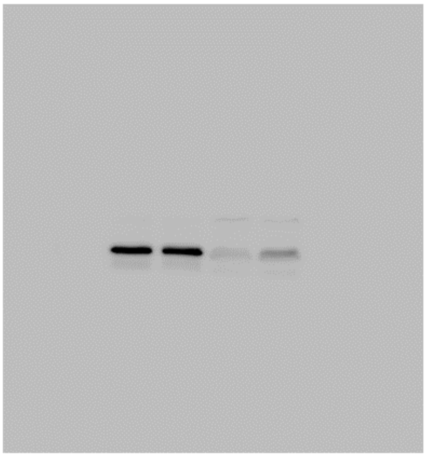

Figure S5F-GPX4

Antibody information: Abcam, ab41787, 1:1000

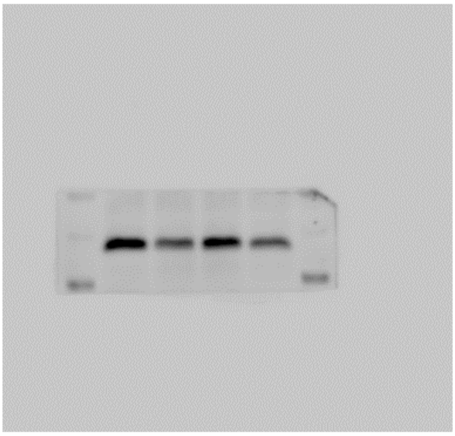

Figure S5F-OTUD5

Antibody information: Cell signaling, 20087S, 1:1000

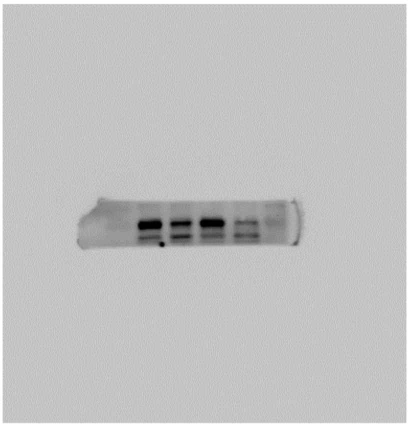

Figure S5F-Actin

Antibody information: Cell Signaling, 4970S, 1:1000

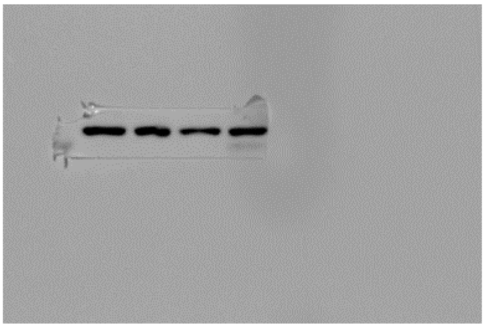

Uncropped immunoblot gels for Supplementary Figure 6

Figure S6A-mTOR

Antibody information: Cell signaling, 2983S, 1:1000

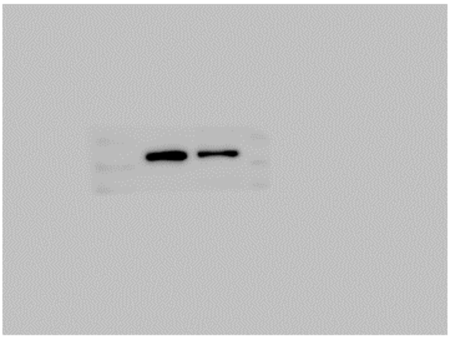

Figure S6A-mTOR-Ser2448

Antibody information: Cell signaling, 5536S, 1:500

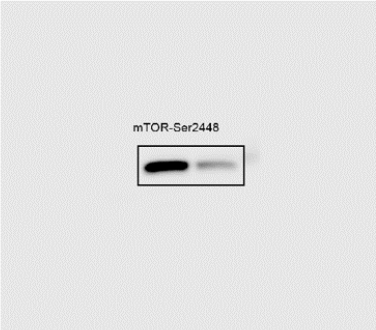

Figure S6A-mTOR-Ser2481

Antibody information: Cell signaling, 2974S, 1:500

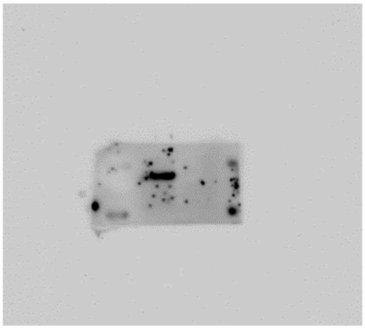

Figure S6A-S6K

Antibody information: Cell signaling, 9202S, 1:1000

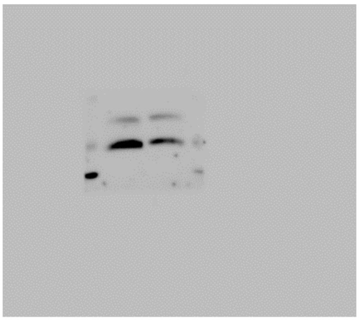

Figure S6A-S6K- Ser371

Antibody information: Cell signaling, 9208S, 1:500

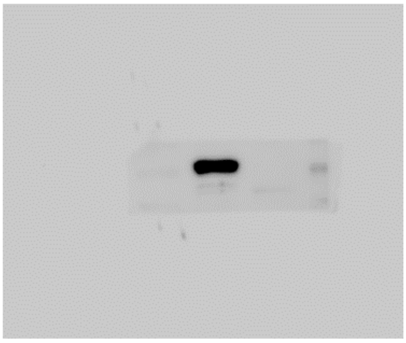

Figure S6A- Actin

Antibody information: Cell Signaling, 4970S, 1:1000

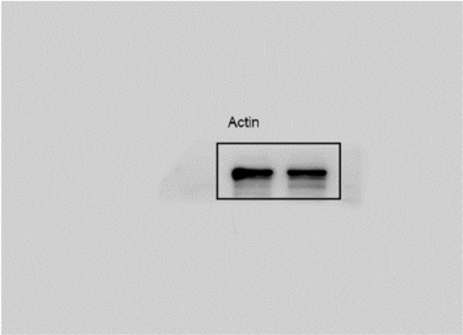

Uncropped immunoblot gels for Supplementary Figure 7

Figure S7A-Flag

Antibody information: Cell Signaling, 14793S, 1:1000

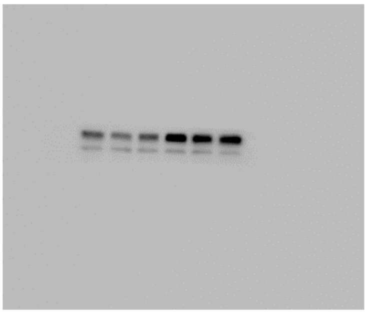

Figure S7A-GPX4

Antibody information: Abcam, ab125066, 1:1000

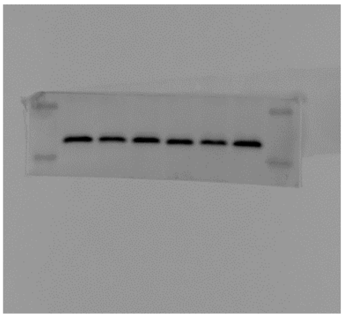

Figure S7A-Actin

Antibody information: Cell Signaling, 4970S, 1:1000

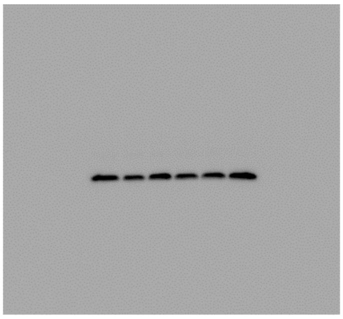

Figure S7c-Flag

Antibody information: Cell Signaling, 14793S, 1:1000

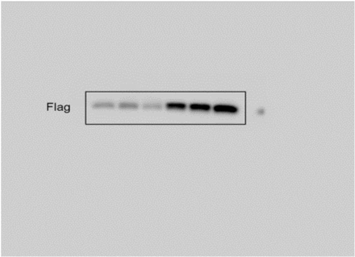

Figure S7c -GPX4

Antibody information: Abcam, ab125066, 1:1000

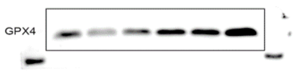

Figure S7c-S6K

Antibody information: Cell signaling, 9202S, 1:1000

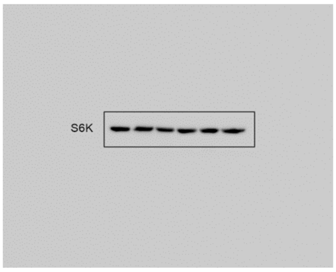

Figure S7c-p-S6K

Antibody information: Cell signaling, 9208S, 1:500

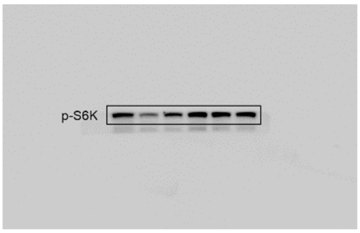

Figure S7c-4-HNE

Antibody information: Abcam, ab48506, 1:500

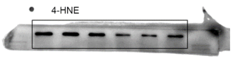

Figure S7c-LC3

Antibody information: Cell Signaling, 4108S, 1:1000

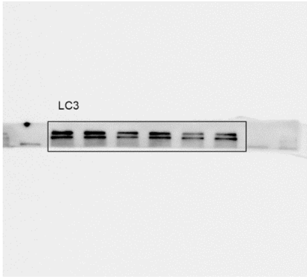

Figure S7c-ACTIN

Antibody information: Cell Signaling, 4970S, 1:1000

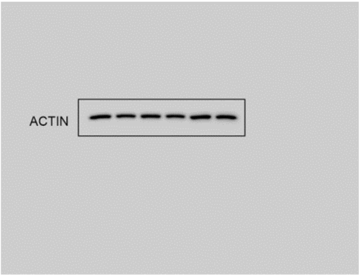

Figure S7c-IP-GPX4

Antibody information: Santa Cruz Biotechnology, Sc-166437, 2 μg per 500 μg protein

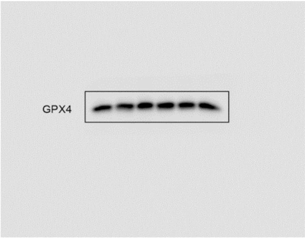

Figure S7c-IP-Ubi

Antibody information: Cell Signaling, 20326S, 1:1000

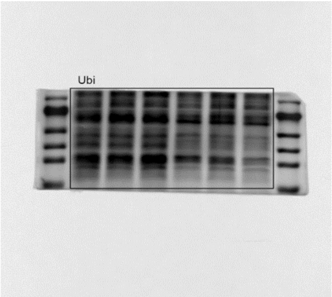

Supplement: Supplementary file 1 — Supplementary Information [file 41467_2023_44228_MOESM1_ESM.pdf]
