# Supplementary material for: Autophagy of OTUD5 destabilizes GPX4 to confer ferroptosis-dependent kidney injury
Source: Nat Commun. 2023 Dec 18;14:8393. doi: 10.1038/s41467-023-44228-5 (PMC10728081; doi:10.1038/s41467-023-44228-5)
Supplement: Supplementary file 3 — Reporting Summary [file 41467_2023_44228_MOESM3_ESM.pdf]

nature portfolio

Corresponding author(s): Jun Liu, Xiang-Ming Yan&Ching-Hsien Chen

Last updated by author(s): Nov 30, 2023

Reporting Summary

Nature Portfolio wishes to improve the reproducibility of the work that we publish. This form provides structure for consistency and transparency in reporting. For further information on Nature Portfolio policies, see our Editorial Policies and the Editorial Policy Checklist.

Statistics

For all statistical analyses, confirm that the following items are present in the figure legend, table legend, main text, or Methods section.

n/a	Confirmed	
☐	☒	The exact sample size (*n*) for each experimental group/condition, given as a discrete number and unit of measurement
☐	☒	A statement on whether measurements were taken from distinct samples or whether the same sample was measured repeatedly
☐	☒	The statistical test(s) used AND whether they are one- or two-sided<br>*Only common tests should be described solely by name; describe more complex techniques in the Methods section.*
☒	☐	A description of all covariates tested
☒	☐	A description of any assumptions or corrections, such as tests of normality and adjustment for multiple comparisons
☐	☒	A full description of the statistical parameters including central tendency (e.g. means) or other basic estimates (e.g. regression coefficient) AND variation (e.g. standard deviation) or associated estimates of uncertainty (e.g. confidence intervals)
☐	☒	For null hypothesis testing, the test statistic (e.g. *F*, *t*, *r*) with confidence intervals, effect sizes, degrees of freedom and *P* value noted<br>*Give P values as exact values whenever suitable.*
☒	☐	For Bayesian analysis, information on the choice of priors and Markov chain Monte Carlo settings
☒	☐	For hierarchical and complex designs, identification of the appropriate level for tests and full reporting of outcomes
☒	☐	Estimates of effect sizes (e.g. Cohen's *d*, Pearson's *r*), indicating how they were calculated

Our web collection on statistics for biologists contains articles on many of the points above.

Software and code

Policy information about availability of computer code

Data collection	Flow cytometry data were acquired by a Beckman Coulter Flow Cytometer (version: Gallios); Western blot data were acquired by ibright imaging-systems (version: FL1500); Immunofluorescence and pathological staining images were acquired by OLYMPUS microscope (version: CX23); ELISA data were acquired by Magellan Standard (Versions 7.2);
Data analysis	For data analysis, GraphPad Prism (version 9) and R (Versions 4.3.0) were used. Flow cytometry data were analyzed by Flow Jo (version 10.8.1). Western Blot, immunofluorescence and pathological staining images were analysed by Image J Software(Version: 1.51). R scripts used in this study are available on GitHub (https://github.com/Toby111/scRNA-spatial-code.git)

For manuscripts utilizing custom algorithms or software that are central to the research but not yet described in published literature, software must be made available to editors and reviewers. We strongly encourage code deposition in a community repository (e.g. GitHub). See the Nature Portfolio guidelines for submitting code & software for further information.

Data

Policy information about availability of data

All manuscripts must include a data availability statement. This statement should provide the following information, where applicable:

- Accession codes, unique identifiers, or web links for publicly available datasets
- A description of any restrictions on data availability
- For clinical datasets or third party data, please ensure that the statement adheres to our policy

Source data for Figures 1e, g, i, 1j, 2a, d, e, g-j, 3b-h, 4a-h, 5e-i, 6b-g, 7b-h, S1a, b, i, k, l, S3c, S4a, c, S5b, S6b, c and S7a-c are provided as source data file. An

uncropped image of the Western Blot and flow cytometric gating strategies are provided in Souce data file. single-cell RNA and sequencing and spatial transcriptomics data have been deposited under GSE244330 (https://www.ncbi.nlm.nih.gov/geo/query/acc.cgi?acc=GSE244330).

Research involving human participants, their data, or biological material

Policy information about studies with human participants or human data. See also policy information about sex, gender (identity/presentation), and sexual orientation and race, ethnicity and racism.

Reporting on sex and gender	n/a
Reporting on race, ethnicity, or other socially relevant groupings	n/a
Population characteristics	n/a
Recruitment	n/a
Ethics oversight	n/a

Note that full information on the approval of the study protocol must also be provided in the manuscript.

Field-specific reporting

Please select the one below that is the best fit for your research. If you are not sure, read the appropriate sections before making your selection.

- [x] Life sciences
- [ ] Behavioural & social sciences
- [ ] Ecological, evolutionary & environmental sciences

For a reference copy of the document with all sections, see nature.com/documents/nr-reporting-summary-flat.pdf

Life sciences study design

All studies must disclose on these points even when the disclosure is negative.

Sample size	Sample size is indicated in the respective figure legends. The sample size was chosen to generate significant statistical differences between groups. No statistical method was used to predetermine the sample size. In vitro experiments were repeated at least three times. For in vivo experiments, our sample size is at least 5 mice per group, which is determined based on previous experience and standards in the field and by the availability of knock out mice as well as the littermates animals.
Data exclusions	The study exclude no experimental data intentionally when positive and negative controls indicating that the experiment worked with reasonable standard errors.
Replication	All data were performed at least in triplicate independently with similar results. All data were the results from at least three biological or technical repeats.
Randomization	Mice, tissue and cell lines were randomly into the different groups.
Blinding	Investigators were blinded to group identity during data collection and analysis.

Reporting for specific materials, systems and methods

We require information from authors about some types of materials, experimental systems and methods used in many studies. Here, indicate whether each material, system or method listed is relevant to your study. If you are not sure if a list item applies to your research, read the appropriate section before selecting a response.

Materials & experimental systems

n/a	Involved in the study	
[ ]	[x]	Antibodies
[ ]	[x]	Eukaryotic cell lines
[x]	[ ]	Palaeontology and archaeology
[ ]	[x]	Animals and other organisms
[x]	[ ]	Clinical data
[x]	[ ]	Dual use research of concern
[x]	[ ]	Plants

Methods

n/a	Involved in the study	
[x]	[ ]	ChIP-seq
[ ]	[x]	Flow cytometry
[x]	[ ]	MRI-based neuroimaging

Antibodies

Antibodies used

Anti-Glutathione Peroxidase 4, supplier: Abcam, catalog number: ab125066, clone number: EPNCIR144
Anti-Glutathione Peroxidase 4, supplier: Abcam, catalog number: ab41787
Anti-4 Hydroxynonenal, supplier: Abcam, catalog number: ab48506, clone number: HNEJ-2
Anti-F4/80, supplier: Abcam, catalog number: ab300421, clone number: EPR26545-166
Anti-OTUD5 Polyclonal Antibody, supplier: Invitrogen, catalog number: PA5-20611
Anti-OTUD5 Polyclonal Antibody, supplier: Proteintech, catalog number: 21002-1-AP
Anti-OTUD5 Rabbit mAb, supplier: Cell Signaling, catalog number: 20087S, clone number: D8Y2U
Anti-GPX4 Antibody supplier: Cell Signaling, catalog number: 52455S
Anti-p70 S6 Kinase Antibody, supplier: Cell Signaling, catalog number: 9202S
Anti-Phospho-p70 S6 Kinase (Ser371) Antibody, supplier: Cell Signaling, catalog number: 9208S
Anti-mTOR Rabbit mAb, supplier: Cell Signaling, catalog number: 2983S, clone number: 7C10
Anti-Phospho-mTOR (Ser2481) Antibody supplier: Cell Signaling, catalog number: 2974S
Anti-Phospho-mTOR (Ser2448) Rabbit mAb, supplier: Cell Signaling, catalog number: 5536S, clone number: D9C2
Anti-DYKDDDDK Tag Rabbit mAb, supplier: Cell Signaling, catalog number: 14793S, clone number: D6W5B
Anti-Ubiquitin Mouse mAb, supplier: Cell Signaling, catalog number: 3936S, clone number: P4D1
Anti-Ubiquitin Rabbit mAb, supplier: Cell Signaling, catalog number: 20326S, clone number: E6K4Y
Anti-LC3A/B Antibody, supplier: Cell Signaling, catalog number: 4108S
Anti-Beclin-1 Antibody, supplier: Cell Signaling, catalog number: 3738S
Anti-Atg5 Rabbit mAb, supplier: Cell Signaling, catalog number: 9980S, clone number: D5G3
Anti-Hamartin/TSC1, supplier: Cell Signaling, catalog number: 6935S, clone number: D43E2
Anti-LAMP1 Rabbit mAb, supplier: Cell Signaling, catalog number: 9091S, clone number: D2D11
Anti-OTUB1 Rabbit mAb, supplier: Cell Signaling, catalog number: 3783S, clone number: D8F7
Anti-UBR5 Rabbit mAb, supplier: Cell Signaling, catalog number: 65344S, clone number: D6O8Z
Anti-XIAP Antibody, supplier: Cell Signaling, catalog number: 2042S
Anti-TRIM21 Rabbit mAb, supplier: Cell Signaling, catalog number: 92043S, clone number: D1O1D
Anti-mouse IgG, HRP-linked Antibody, supplier: Cell Signaling, catalog number: 7076S
Anti-rabbit IgG, HRP-linked Antibody, supplier: Cell Signaling, catalog number: 7074S
Anti-β-actin Rabbit mAb, supplier: Cell Signaling, catalog number: 4970S, clone number:13E5
Anti-HSPA8/HSC70, supplier: Santa Cruz Biotechnology, catalog number: sc-7298, clone number: B-6
Anti-VSP4, supplier: Santa Cruz Biotechnology, catalog number: sc-133122, clone number: E-8
Anti-Glutathione Peroxidase 4, supplier: Santa Cruz Biotechnology, catalog number: sc-166437, clone number: D-3
Anti-KIM-1 (HAVCR1) supplier: Boster Biological Technology, catalog number: BA3536
Normal rabbit IgG, supplier: Santa Cruz Biotechnology, catalog number: sc-2027
Normal mouse IgG, supplier: Santa Cruz Biotechnology, catalog number: sc-2025

Validation

All antibodies used in this study are commercially available and validated by the vendor for the species and assay. Specific validation information is available on the website from the vendors and listed below:
Anti-Glutathione Peroxidase 4, https://www.abcam.cn/products/primary-antibodies/glutathione-peroxidase-4-antibody-epncir144-ab125066
Anti-Glutathione Peroxidase 4, https://www.abcam.cn/products/primary-antibodies/glutathione-peroxidase-4-antibody-ab41787
Anti-4 Hydroxynonenal, https://www.abcam.cn/products/primary-antibodies/4-hydroxynonenal-antibody-hnej-2-ab48506.html
Anti-F4/80, https://www.abcam.cn/products/primary-antibodies/f480-antibody-epr26545-166-ab300421
Anti-OTUD5 Polyclonal Antibody, https://www.thermofisher.cn/cn/zh/antibody/product/OTUD5-Antibody-Polyclonal/PA5-20611
Anti-OTUD5 Polyclonal Antibody, https://www.thermofisher.cn/cn/zh/antibody/product/OTUD5-Antibody-Polyclonal/21002-1-AP
Anti-OTUD5 Rabbit mAb, https://www.cellsignal.cn/products/primary-antibodies/otud5-d8y2u-rabbit-mab/20087
Anti-GPX4 Antibody, https://www.cellsignal.cn/products/primary-antibodies/gpx4-antibody/52455
Anti-p70 S6 Kinase Antibody, https://www.cellsignal.com/products/primary-antibodies/p70-s6-kinase-antibody/9202
Anti-Phospho-p70 S6 Kinase(Ser371)Antibody, https://www.cellsignal.com/products/primary-antibodies/phospho-p70-s6-kinase-ser371-antibody/9208
Anti-mTOR Rabbit mAb, https://www.cellsignal.cn/products/primary-antibodies/mtor-7c10-rabbit-mab/2983
Anti-Phospho-mTOR(Ser2481)Antibody, https://www.cellsignal.com/products/primary-antibodies/phospho-mtor-ser2481-antibody/2974
Anti-Phospho-mTOR(Ser2448)Rabbit mAb, https://www.cellsignal.com/products/primary-antibodies/phospho-mtor-ser2448-d9c2-xp-rabbit-mab/5536
Anti-DYKDDDDK Tag Rabbit mAb, https://www.cellsignal.cn/products/primary-antibodies/dykddddk-tag-d6w5b-rabbit-mab-binds-to-same-epitope-as-sigma-s-anti-flag-m2-antibody/14793
Anti-Ubiquitin Mouse mAb, https://www.cellsignal.cn/products/primary-antibodies/ubiquitin-p4d1-mouse-mab/3936
Anti-Ubiquitin Rabbit mAb, https://www.cellsignal.com/products/primary-antibodies/ubiquitin-e6k4y-xp-rabbit-mab/20326
Anti-LC3A/B Antibody, https://www.cellsignal.com/products/primary-antibodies/lc3a-b-antibody/4108
Anti-Beclin-1 Antibody, https://www.cellsignal.com/products/primary-antibodies/beclin-1-antibody/3738
Anti-Atg5 Rabbit mAb, https://www.cellsignal.cn/products/primary-antibodies/atg5-d5g3-rabbit-mab/9980
Anti-Hamartin/TSC1, https://www.cellsignal.com/products/primary-antibodies/hamartin-tsc1-d43e2-rabbit-mab/6935
Anti-LAMP1 Rabbit mAb, https://www.cellsignal.com/products/primary-antibodies/lamp1-d2d11-xp-rabbit-mab/9091
Anti-OTUB1 Rabbit mAb, https://www.cellsignal.com/products/primary-antibodies/otub1-d8f7-rabbit-mab/3783
Anti-UBR5 Rabbit mAb, https://www.cellsignal.com/products/primary-antibodies/ubr5-d6o8z-rabbit-mab/65344
Anti-XIAP Antibody, https://www.cellsignal.cn/products/primary-antibodies/xiap-antibody/2042

Anti-TRIM21 Rabbit mAb, https://www.cellsignal.cn/products/primary-antibodies/trim21-d1o1d-rabbit-mab/92043
Anti-mouse IgG HRP-linked Antibody, https://www.cellsignal.cn/products/secondary-antibodies/anti-mouse-igg-hrp-linked-antibody/7076
Anti-rabbit IgG HRP-linked Antibody, https://www.cellsignal.cn/products/secondary-antibodies/anti-rabbit-igg-hrp-linked-antibody/7074
Anti-β-actin Rabbit mAb, https://www.cellsignal.com/products/primary-antibodies/b-actin-13e5-rabbit-mab/4970
Anti-HSPA8/HSC70, https://www.scbt.com/p/hsc-70-antibody-b-6/
Anti-VSP4, https://www.scbt.com/p/vps4-antibody-e-8
Anti-Glutathione Peroxidase 4, https://www.scbt.com/zh/p/gpx-4-antibody-d-3
Anti-KIM-1 (HAVCR1), https://www.boster.com.cn/index/products/productsDetail?goods_sn=BA3536
Normal rabbit IgG, https://www.scbt.com/p/normal-mouse-igg
Normal mouse IgG, https://www.scbt.com/zh/p/normal-mouse-igg

Eukaryotic cell lines

Policy information about cell lines and Sex and Gender in Research

Cell line source(s)	HK2 (catalog number: CRL-2190) and HEK293T (catalog number: CRL-3216) cell line were purchased from the American Type Culture Collection; Mouse primary renal tubular cells (PRTCs) were isolated from both male and female mice.
Authentication	None of the cell line used in this study were authenticated.
Mycoplasma contamination	All cell line used in this study were tested negative for mycoplasma contamination.
Commonly misidentified lines (See ICLAC register)	No commonly misidentified cell line were used in this study.

Animals and other research organisms

Policy information about studies involving animals; ARRIVE guidelines recommended for reporting animal research, and Sex and Gender in Research

Laboratory animals	4 to 6-week-old wild type C57BL/6 mice; 4 to 6-week-old Otud5-floxed mice on a C57BL/6 genetic background; 4 to 6-week-old Pax8-Cre on a C57BL/6 genetic background; All mice were housed in a pathogen-free environment with a temperature of 22℃, a light/dark cycle of 12h/12h, and relative humidity of 50-60%.
Wild animals	No wild animals were used in this study.
Reporting on sex	Both sex mice were used in this study indiscriminately.
Field-collected samples	This study include no field-collected samples
Ethics oversight	This study was approved by the Institutional Review Board of the Children's Hospital of Soochow University. All experimental procedures were conducted following the guidelines of the Institutional Animal Care and Use Committee (IACUC) of the Children's Hospital of Soochow University.

Note that full information on the approval of the study protocol must also be provided in the manuscript.

Flow Cytometry

Plots

Confirm that:

- [x] The axis labels state the marker and fluorochrome used (e.g. CD4-FITC).
- [x] The axis scales are clearly visible. Include numbers along axes only for bottom left plot of group (a 'group' is an analysis of identical markers).
- [x] All plots are contour plots with outliers or pseudocolor plots.
- [x] A numerical value for number of cells or percentage (with statistics) is provided.

Methodology

Sample preparation	Cells were collected and washed twice with pre-chilled 1×PBS. The cells were then resuspended in staining buffer (420201, BioLegend) and stained with BODIPY 581/591 C11 at room temperature for 30 minutes. After washing and staining with 7-AAD, the cells were resuspended in cell staining buffer and subjected to flow cytometry analysis.
Instrument	Beckman Coulter Gallios Flow Cytometer, 3 lasers, 10 channels
Software	FlowJo (V10.8.1)

Cell population abundance	The abundance was over 90% on post sort-checks.
Gating strategy	Cells were gated by FSH/SSA gates to select single cells. Cells were then gated by FL1 and FL3 to discriminate between live and ferroptosis cells using BODIPY 581/591 C11 and 7-AAD. The gating strategy is provided in the Supplementary figures.

☒ Tick this box to confirm that a figure exemplifying the gating strategy is provided in the Supplementary Information.
